# Where, When and Why Do Tsetse Contact Humans? Answers from Studies in a National Park of Zimbabwe

**DOI:** 10.1371/journal.pntd.0001791

**Published:** 2012-08-28

**Authors:** Stephen J. Torr, Andrew Chamisa, T. N. Clement Mangwiro, Glyn A. Vale

**Affiliations:** 1 Natural Resources Institute, University of Greenwich, Chatham, United Kingdom; 2 Division of Tsetse Control, Harare, Zimbabwe; 3 Bindura University of Science Education, Bindura, Zimbabwe; 4 Southern African Centre for Epidemiological Modelling and Analysis, University of Stellenbosch, Stellenbosch, South Africa; Universidad de Buenos Aires, Argentina

## Abstract

**Background:**

Sleeping sickness, also called human African trypanosomiasis, is transmitted by the tsetse, a blood-sucking fly confined to sub-Saharan Africa. The form of the disease in West and Central Africa is carried mainly by species of tsetse that inhabit riverine woodland and feed avidly on humans. In contrast, the vectors for the East and Southern African form of the disease are usually savannah species that feed mostly on wild and domestic animals and bite humans infrequently, mainly because the odours produced by humans can be repellent. Hence, it takes a long time to catch many savannah tsetse from people, which in turn means that studies of the nature of contact between savannah tsetse and humans, and the ways of minimizing it, have been largely neglected.

**Methodology/Principal Findings:**

The savannah tsetse, *Glossina morsitans morsitans* and *G. pallidipes*, were caught from men in the Mana Pools National park of Zimbabwe. Mostly the catch consisted of young *G. m. morsitans*, with little food reserve. Catches were increased by 4–8 times if the men were walking, not stationary, and increased about ten times more if they rode on a truck at 10 km/h. Catches were unaffected if the men used deodorant or were baited with artificial ox odour, but declined by about 95% if the men were with an ox. Surprisingly, men pursuing their normal daily activities were bitten about as much when in or near buildings as when in woodland. Catches from oxen and a standard ox-like trap were poor indices of the number and physiological state of tsetse attacking men.

**Conclusion/Significance:**

The search for new strategies to minimize the contact between humans and savannah tsetse should focus on that occurring in buildings and vehicles. There is a need to design a man-like trap to help to provide an index of sleeping sickness risk.

## Author Summary

To identify where to look for new strategies to limit the contact between tsetse and humans, and so reduce the transmission of sleeping sickness, we examined various factors influencing the numbers of tsetse caught from men. Catches were increased if the men walked, or travelled a little faster on a truck, and were reduced if an ox were nearby. There was no effect of making the men smell like an ox, or of spraying the men with deodorant. Intriguingly, while it is usually assumed tacitly that most human/tsetse contact occurs when people enter the normal woodland habitat of the flies, we found that just as much contact occurred due to tsetse visiting human habitations. We also operated standard ox-like traps to assess whether their catches could be used as indices of the biting rate on humans, but found too many distinctions between catches from these traps and people. Overall, it appeared that the search for means of restricting human/fly contact should focus on that occurring in buildings and vehicles, and that attempts should be made to design traps that are more man-like.

## Introduction

At least 90% of cases of sleeping sickness, or human African trypanosomiasis (HAT), are caused by *Trypanosoma brucei gambiense* transmitted by tsetse flies, particularly *Glossina fuscipes* and *Glossina palpalis* that inhabit riverine woodland [1]. This form of the disease, called Gambian sleeping sickness, occurs across much of Central and West Africa. Savannah tsetse, such as *G. morsitans morsitans* and *G. pallidipes*, transmit *Trypanosomas brucei rhodesiense* which causes Rhodesian sleeping sickness, a more acute disease typically associated with wilderness areas in East and Southern Africa; this disease is a zoonosis, involving wild and domestic animals as important hosts for tsetse and trypanosomes [2].

Over the last decade, the annual number of sleeping sickness cases reported across Africa has decreased to<10,000 cases, largely due to concerted efforts to detect and treat cases of Gambian sleeping sickness [1]. However, where Rhodesian sleeping sickness foci occur in wilderness areas, the number of cases has not declined (*e.g*., Tanzania [3]; see also national figures between 1997 and 2006 for Malawi and Zambia [4]). Moreover, the number of cases in non-endemic countries has also increased over the same period, perhaps due partly to increasing tourism to endemic situations – the tourists usually having good access to diagnostic facilities [5], [6]. In contrast, indigenous people at risk are typically scattered in areas where medical centres are few and rudimentary. No countries have a national screening programme, so that while no more than a few hundred cases of Rhodesian sleeping sickness are actually reported per year, the true number of cases occurring is perhaps ten times greater [7].

There are two reasons why Rhodesian sleeping sickness persists. First, the important reservoir hosts – wild mammals – cannot be readily treated with drugs to control trypanosomes, nor with insecticides to control tsetse. Second, campaigns to eliminate tsetse from extensive wilderness areas by, say, aerial spraying can be ecologically unacceptable there, and are affordable only to the richer nations [8]. Hence, the current ability to protect indigenous populations and visitors against Rhodesian sleeping sickness is poor. We need innovative strategies that reduce the risk of being bitten through an improved understanding of man/tsetse contact. For example, where and when is transmission most likely to occur, and which sex and species of tsetse might be most involved? Are there are any particular circumstances associated with enhanced transmission of HAT, and if so how could they be minimized.

Hitherto, research on savannah tsetse has been concerned with reducing the extensive economic damage caused by animal trypanosomiasis [9]. Consequently, research on fly/host contact has involved analysis of the responses of tsetse to cattle, largely to identify the most effective stimuli to use in artificial baits for tsetse control [10], or to study the efficacy of insecticide applied to cattle [11]. Little attention was given to human baits in these contexts since humans have long been known as strongly repellent to tsetse of most ages and physiological states [12], [13]. To redress this gap in our knowledge, we undertook field studies in Zimbabwe to compare the numbers and physiological condition of flies caught from men and other baits under a variety of circumstances.

## Materials and Methods

### Ethical statement

The procedures for sampling tsetse attracted to humans and livestock followed long-standing protocols practiced at Rekomitjie and received ethical approval from the Division of Tsetse Control, Zimbabwe, and the University of Greenwich (Project ref. B0203), UK. All subjects involved in this study were staff employed by the Division of Tsetse Control, Government of Zimbabwe. Before recruitment, the Division explains the risks associated with tsetse, other disease vectors and wild animals, and of the social hardships of working on a remote field station. On recruitment, personnel sign a document indicating their informed consent to work under such conditions. This document is held at the offices of the Division of Tsetse Control. In the 53 years of research at Rekomitjie, no case of sleeping sickness has been found to be contracted there, despite the good diagnostic facilities of the station. Cattle kept at Rekomitjie are protected by trypanocides [14].

### Study area

Most studies were performed within 2 km of Rekomitjie Research Station (16°10′S, 29°25′E, altitude 503 m) where *G. m. morsitans* and *G. pallidipes* are abundant. The station is in the Mana Pools National Park of the Zambezi Valley, close to the escarpment that defines the valley, and about 50 km from the main tourist locations beside the Zambezi River. About 60 people live on station, and many wild animals, including warthog, kudu, elephant and buffalo are common in the area. Four seasons were recognized: 1) hot-dry (Sep-Nov), with a mean daily maximum temperature of 35.8°C during the present study and a total rainfall of 77 mm, 2) warm-wet (Dec-Feb), 32.8°C , 523 mm, 3) cool-damp (Mar-May), 31.6°C , 74 mm, and 4) cold-dry (Jun-Aug), 28.5°C, 0 mm.

### Attraction of tsetse to humans

Flies on the men were caught by handnets as they landed. The men were African, of medium build, in a variety of light-weight civilian clothing but often in green overalls worn immediately next to the skin. Such attires were typical of the station's workforce, on and off duty. The individuals used in each experiment varied according to the persons available, but since there was no evidence of distinctive responses to particular individuals the data for all individuals were pooled.

### General assessment of biting risk

To obtain a sample of flies responding to humans, across the day in various habitats, two men operated for 6–8 hours per day between 06.00 h and 18.00 h, for 5–19 (mean 16) days per month, in each of the 14 months between August 2009 and September 2010. Only flies attempting to probe the humans were caught. The men followed closely the normal activities of the station's work force, spending their time about equally between: (i) savannah woodland, (ii) the mainly cleared area (∼30ha) of the research station's grounds, and (iii) inside the station buildings. Most of these structures consisted of several rooms and most were fully walled, but some were only partially so. Roofs were of thatch, or corrugated sheets of galvanized iron or asbestos, the latter two materials being mostly above a ceiling that kept the rooms cool. Doors and windows were open or closed as per the normal policy of the buildings' occupants, but were mostly open by day and closed at night.

### Specific factors in risk

A series of experiments assessed the effect of various factors on catches from men. In these studies the number of flies around the baits was often larger than in the general study, above, making for inefficient catching if the men concentrated on each fly to see whether it eventually probed. Hence, catches consisted of all tsetse landing on the men, whether the flies were probing or not.

#### Effect of locomotion

Responses of tsetse to mobile men were studied by the fly-round technique [15]. For the “standard” fly-round, three men walked at *∼*3 km/h along a path, 3 km long through woodland, in the last 2.5 h before sunset. The path was divided into 100 m sections each of which was completed in 2 min of walking with a 3 min stop. To compare these catches with those from stationary men, on days randomly interspersed between the days when the standard fly-round was used, the men were stationed at one position on the path – the particular position being changed between days so that in the course of the study the sampling covered various points evenly spaced along the whole path.

#### Effect of an accompanying ox or motor vehicle

Sometimes the mobile and stationary men were accompanied by a brown cross-breed ox of ∼400 kg, and sometimes by a maroon pick-up truck with a twin cab (Toyota Hi-Lux). For the mobile truck, two men sat on the open back, and the third was in the cab with the driver while traveling at 5 km/h between the stations, and they got off to be beside the vehicle at the stops. At the stationary truck, the men were in various positions in and out of it. No attempt was made to catch tsetse from the truck itself since it was too large to inspect conveniently when mobile. However, flies were caught from the ox with the men, and were recorded separately. Any flies on the men of the men + ox combination received first attention for catching.

#### Effect of speed

During the above work , the mobile truck was operated like mobile men alone, *i.e.*, moving 100 m and then stopping to compete five minutes per fly-round sector, so that its average speed was only 1.2 km/h. However, it was suspected that if the truck were operated at greater speed, more typical of its use on the station, then more tsetse might be attracted [16] thereby increasing the biting risk. To test this hypothesis, the truck was operated for 30 min of continuous travel at 10 km/h between 0630 h and 0700 h, and 1715 and 1745 h on the tracks around Rekomitjie, for eight days in November 2010. At the same time, the standard fly-round was operated on a nearby path 600 m long, *i.e.*, six sectors, with a change of path each day. Another type of fly-round was operated similarly, except that the men walked continuously, covering about 1200 m and travelling at an average speed that was double that of the standard fly-round.

#### Effect of ox odour

Sometimes one of the men in the party carried odour dispensers [17] baited with a blend of chemicals which are naturally present in ox odour. The blend, called AOP, comprised acetone (100 mg/h), 1-octen-3-ol (0.5 mg/h), 3*n*-propyl phenol (0.1 mg/h) and 4-methyl phenol (1.0 mg/h).

#### Masking human odours

Given that the repellence of men is known to be due largely to odour from their skin [18], attempts were sometimes made to deodorize their skin. About 15 min before acting as baits, the men washed their whole bodies with carbolic soap and then sprayed themselves all over with deodorant aerosol (Shield, Unilever Ltd, Zimbabwe) that contained aluminium chlorohydrate as the active constituent. All of the clothing that the men then put on, ie, overalls, underpants, socks, shoes and hats, had been freshly laundered with detergent.

For each of the separate experiments used to address the above four matters, various baits were allocated at random to separate days in blocks of days. The standard fly-round, involving walking men with no treatment, was incorporated in each of the separate experiments. Hence, when it was required to compare treatments used in different experiments, the mean catches of these treatments were expressed as a proportion of the mean catch of the standard in their respective experiments, to give an index of efficacy that was independent of changes in the availabilities of flies from one experiment to another.

#### Catches from traps

At the same time as the studies with human baits, stationary Epsilon traps [19] baited with AOP were placed in woodland within 100–200 m of the fly-round path and at least 300 m from any stationary men. Humans have to be continually much closer to traps before interfering materially with the catches [20].

#### Catches at a traffic barrier

The pick-up truck, above, was the only vehicle available for the Rekomitjie studies, so that it was impossible to assess there the extent to which the response to this truck was typical of that to vehicles in general. However, data to elucidate this matter were available from a traffic barrier, operated 25 km West of Rekomitjie by the Division of Tsetse Control. At this barrier the flies in vehicles are routinely caught by handnets and removed to prevent flies being taken to uninfested parts of the Zimbabwe high-veld. The barrier (16°11′S, 29°9′E) is at the junction of the untarred road from the station and tourist camps of the Mana Pools park and the main tarred road that runs North-South through the Chirundu Border Post on the Zambezi, to connect Harare and Lusaka, *i.e.*, the capitals of Zimbabwe and Zambia, respectively. In the Zambezi valley, before being stopped at the barrier, the South-bound vehicles on each road passed through similar types of woodland, heavily infested with both species of tsetse, although the speed limit of 120 km/h on the tarred road is at least treble the speed that vehicles usually reach on the untarred one. Attendants at the barrier recorded the numbers of tsetse caught and whether the vehicle originated from the tarred or untarred road. No note of the type of vehicle was kept, but since the barrier stopped all traffic, including large lorries, saloon cars, pick-ups, and towed caravans, the catches indicated the samples of tsetse to which people are exposed while in and on the generality of vehicles, although humans may catch and discard tsetse inside the vehicles before reaching the barrier.

### Physiological status of tsetse

Tsetse caught by handnets at Rekomitjie were recorded according to whether they were settled on a vertical surface with their head uppermost or downmost, it being known that the head-up orientation is typical of flies with depleted food reserves and motivated to feed rather than mate [15]. If the flies were on a horizontal surface, such as the shoulder, so that any up/down orientation could not be identified, the flies were recorded as head-horizontal.

Catches by traps, or by handnets from humans or the ox, were examined for their ovarian condition [21] and wing fray [22] as indices of age. Males had their wings removed for fray assessment and were then dried and weighed before and after extraction of fat by three 24 h baths in chloroform. The fatless abdomen was then removed and weighed. These three weights allowed calculation of the following measurements.

1. TW: fatless dry weight of the thorax, legs and head, as a measure of fly size.
2. Fat%: fat content of the fly as a percent of TW, giving an indication of energy reserves.
3. AB%: fatless dry weight of the abdomen as a percent of TW; this comprises the weight of the abdominal tissues plus any remnant of undigested blood-meal.

### Statistics

Statistical analysis of the daily catches was made by *F*- or t-tests after transformation to log(n+1). Discussion of the mean catches refers to the detransformed means. Heterogeneity of catch compositions was examined by chi-squared. The term “significant” implies *P*<0.05. Where sub-samples are pooled there was no significant difference between them, unless stated otherwise.

## Results

### General assessment of biting risk

In the whole sampling period of 221 days, a total of 264 tsetse probing men were caught when the men were in the woodland, as against 189 when near the station buildings and 186 when inside the buildings. This implies that during the normal schedule of human activities on foot, the station's staff was 1.4 times more likely to be bitten in and near buildings rather than in the main woodland habitat of the flies. In all situations the vast majority, *i.e.*, 94–97%, of tsetse were *G. m. morsitans*, the total *G. pallidipes* for all locations combined being only 20 males and 14 females. For *G. m. morsitans* the sex compositions of the catches in the woodland and near the buildings were not significantly different, so the catches at these places were pooled into an “outside” category for comparison with those from inside the buildings (Table 1). At all seasons the percent of females in catches inside was higher, by 2–16 points, than for the combined catches outside, although this effect was significant only in the hot-dry season. Both inside and outside the buildings, the percentage of females was highest in the hot-dry season, being about double that in the cool-damp, but the seasonal effect was significant only for the inside catches.

**Table 1:** Catch of *G. m. morsitans* caught from men inside or outside at various seasons.

Season	Location	Males	Females	% females
**Hot-dry**	Outside	109	59	35.1
	Inside	51	52	50.5
**Warm-wet**	Outside	60	28	31.8
	Inside	10	9	47.4
**Cool-damp**	Outside	77	21	21.4
	Inside	26	8	23.5
**Cold-dry**	Outside	53	23	30.3
	Inside	12	7	36.8
**Total**		398	207	34.2

The men were inside buildings or outside, either near the station buildings or in woodland.

#### Landing site

The body regions on which the flies probed (Table 2) indicated that one of the smallest regions, ie, the hand, was attacked the most, accounting for 28% of catches. Perhaps this was because the hands were never covered with clothing. Considering all body regions, the males of *G. m. morsitans* probed lower on the body than females of this species −27% of males were on the legs and feet vs 14% for females (*P*<0.001). The same trend was observed with *G. pallidipes*, although it was not significant. The main point with the latter species was that both sexes probed mostly low on the body, *i.e.*, 59% of the combined sample for the sexes probed on the legs and feet as against 23% for *G. m. morsitans* (P<0.001). When the flies of either sex or species probed on vertical surfaces, their orientation was always head-up.

**Table 2:** Numbers of tsetse alighting on various parts of men.

*Position*	*G. m. morsitans*		*G. pallidipes*	
	Males	Females	Males	Females
**Head**	33	30	2	1
**Neck**	22	16	0	0
**Shoulder**	52	21	0	0
**Arm**	20	8	0	1
**Hand**	104	65	5	3
**Chest**	6	5	0	2
**Belly**	2	2	0	0
**Back**	50	30	0	0
**Side**	0	0	0	0
**Leg**	62	13	6	5
**Foot**	47	15	7	2
**Total**	398	205	20	14

#### Comparison with standard samples

Despite the above variations in the sex compositions of samples of flies probing men, the over-riding indication was that throughout the sampling period the proportions of females and of *G. pallidipes* in all probing samples were much lower than in trap catches. Of the 21,912 tsetse caught in 233 trap/days, 82.3% were *G. pallidipes*. The proportion of females was 79.0% for *G. pallidipes* and 80.0% for *G. m. morsitans*. Against this, the proportions of *G. pallidipes* and of females from the standard fly-round, involving the capture of all landing flies, not only probers, were somewhat lower than for the probing sample. Of the 2041 tsetse caught in 58 fly-round/days, *G. pallidipes* represented 0.8%, with females forming 17.6% of the *G. pallidipes* and 10.9% of the *G. m. morsitans*.

Although the daily catches from the traps and men differed grossly in their magnitude and composition, trap catches could be useful indices of the biting rate provided they bear a constant relationship to that rate. The catches of *G. pallidipes* probing humans were too low to assess the relationship satisfactorily with this species. The data for *G. m. morsitans* (Table 3) showed that mean daily catches from humans and traps each varied significantly with season, but the seasonal patterns differed markedly. Thus, the catch of flies probing humans, expressed as a percent of trap catches was ∼7 times greater in the hot-dry season than in the cold-dry season.

**Table 3:** Catches of *G. m. morsitan*s from traps and humans.

Season	Trap				Human				(%)
	Days	Trans	SE	Detrans	Days	Trans	SE	Detrans	
**Hot-dry**	66	0.91a	0.054	7.2	59	0.70a	0.028	4.0	56
**Warm-wet**	49	1.00ab	0.068	9.1	51	0.41bc	0.036	1.6	18
**Cool-damp**	38	1.16b	0.070	13.5	51	0.50b	0.033	2.1	16
**Cold-dry**	80	1.18b	0.041	14.1	60	0.34c	0.032	1.2	8

Transformed (Trans) and detransformed (Detrans) mean daily catches are the catches of male and female *G. m. morsitan*s combined from traps or men and the respective standard errors of the transformed means. The mean catches from humans are presented as a percent of that from the traps. In any one column, means not associated with the same letter differ at P<0.05.

### Effects of locomotion, ox, truck and odours

For *G. pallidipes* the catches from the men were too few to show any effect of treatment. For *G. m. morsitans* the salient indications (Table 4) were that none of the treatments increased the catches significantly above those of the standard fly-round. However, catches declined significantly, by 75–86%, when the men were stationary, and by 96–99% when the men were accompanied by an ox. Despite the poor catches from men beside an ox, the catches from the ox they accompanied were large (Table 5), being many times greater than from the men used alone (Table 4, Expt 1), and showing a greater proportion of females and of *G. pallidipes*. Hence, while it seemed that the ox increased the number and variety of tsetse available in the general vicinity of the men, this was more than offset by the ox competing strongly for the flies. For male *G. m. morsitans*, the catches from the mobile ox were five times greater than from the stationary. For female *G. m. morsitans* and male *G. pallidipes*, the difference dropped to only two-fold, and for female *G. pallidipes* there was no significant effect of movement.

**Table 4:** Catches of *G. m. morsitans* from mobile and stationary men in various experiments.

Expt^1^	Mobility	Treatment	Trans	Detrans	Index
**1**	Mobile	Nil (Standard)	1.33a	20.1	1.00
	Mobile	With Ox	0.27c	0.9	0.04
	Stationary	Nil	0.67b	3.7	0.18
	Stationary	With Ox	0.10c	0.3	0.01
		SE	0.060		
**2**	Mobile	Nil (Standard)	1.59a	38.2	1.00
	Mobile	With AOP	1.68a	46.7	1.22
	Stationary	With AOP	1.02b	9.5	0.25
		SE	0.032		
**3**	Mobile	Nil (Standard)	1.69a	47.6	1.00
	Mobile	Toiletries	1.66a	44.5	0.94
	Stationary	Toiletries	0.87b	6.5	0.14
		SE	0.053		
**4**	Mobile	Nil (Standard)	1.43a	26.1	1.00
	Mobile	With truck	1.49a	30.0	1.15
	Stationary	With truck	0.74b	4.5	0.17
		SE	0.063		

Transformed (Trans) and detransformed (Detrains) mean daily catches of male and female *G. m. morsitans* combined, from mobile and stationary men in a number of separate experiments. Means are accompanied by the standard error (SE) of the transformed means, and the index for efficacy, *i.e*., the detransformed mean catch of each treatment expressed as a proportion of the standard. In any one experiment, detransformed means not associated with the same letter differ at *P*<0.05.

AOP = a synthetic blend of acetone, octenol and phenols found in natural host odour. See text for further details.

1 Expt 1: Aug-Dec 2009, 19 replicates. Expt 2: Jan-Apr 2010, 17 replicates. Expt 3: Apr-Jun 2010, 8 replicates. Expt 4: May-Jul 2010, 14 replicates.

**Table 5:** Catches of tsetse from a mobile or stationary ox accompanied by men.

Species	Sex	Mobility	Trans	Detrans
***G. m. morsitans***	Males	Mobile	1.90a	78.1
		Stationary	1.21b	15.2
		SE	0.050	
	Females	Mobile	1.51a	31.5
		Stationary	1.22b	15.6
		SE	0.047	
***G. pallidipes***	Males	Mobile	1.13a	12.4
		Stationary	0.90b	6.9
		SE	0.059	
	Females	Mobile	1.35a	21.3
		Stationary	1.31a	19.4
		SE	0.065	

Transformed (Trans) and detransformed (Detrans) mean daily catches from a mobile or stationary ox with men over 19 replicates of Expt 1, Table 4. Means are accompanied by the standard error (SE) of the transformed means. For any one sex and species, detransformed means not associated with the same letter differ at P<0.05.

#### Effect of speed

In the study to compare the standard fly-round with the continuous fly-round and the truck travelling at the enhanced speed of 10 km/h, the catches of *G. m. morsitans* were too small to show any clear effect of morning vs afternoon on the catches of each sex, and so the catches for both times of day and both sexes were pooled. The resulting detransformed daily means were 1.2 from the men on the standard fly-round, 2.1 for the men moving at greater average speed on the continuous fly-round, and 15.8 for the men on the truck. Only the catches from the truck showed a significant (P<0.001) distinction from either of the other baits. For *G. pallidipes* the catches were smaller, giving eight-day totals of nil from the continuous fly-round, one male from the standard fly-round and five males and 11 females from the men with the truck. The overall pattern of catches with both species was compatible with the indication [12] that the availability of *G. m. morsitans* to mobile baits traveling at 2.0–5.4 km/h is proportional to bait speed.

#### Catches at the traffic barrier

Data for the 48-month period from December 2006 to November 2010 showed the same seasonal pattern in each 12-month period and so they were pooled to display the seasonal effect (Table 6). The number of vehicles originating from the tarred road was always many times greater than from the untarred road, especially in the wetter seasons when the untarred road and most of the tracks feeding into it were poorly passable and closed to the public. However, for *G. m. morsitans* the catch per vehicle on the untarred road was very many times more than that on the tarred road – on average about 200 times more in the hot-dry season and increasing to about 1000 times in the cool-damp, so that the total catches from the untarred road were always by far the greater. Intriguingly, for *G. pallidipes* the catch per vehicle was on average only five times greater on the untarred road, and showed a different seasonal pattern. The upshot was that the proportion of *G. pallidipes* in catches from the untarred road was only 0.3 to 5.6%, as against 28.6 to 70.3% from the tarred road, with by far the highest percent from that road occurring in the hot-dry season.

**Table 6:** Catches of tsetse from vehicles leaving the Zambezi Valley of Zimbabwe at various seasons.

Season	Road	Vehicles	*G. m. morsitans*		*G. pallidipes*	
			Males	Females	Males	Females
**Hot-dry**	Tarred	61197	102	42	160	181
	Untarred	4177	1184	918	40	32
**Warm-wet**	Tarred	55269	23	6	7	10
	Untarred	1218	205	62	3	3
**Cool-damp**	Tarred	45453	7	3	2	2
	Untarred	1265	168	104	10	6
**Cold-dry**	Tarred	41516	29	11	16	25
	Untarred	2448	426	277	0	2
**Total**		212543	2144	1423	238	261

Catches are the total number of tsetse caught from all vehicles passing the traffic barrier at the junction of the tarred road from Chirundu and the untarred road from Mana Pools National Park between December 2006 to November 2010. Each vehicle inspected was recorded as having come from either the untarred road from the Mana Pools National Park or the tarred road from Chirundu.

### Physiological status of tsetse

#### Fly orientation

In all samples of flies caught from men or cattle, the proportion of head-horizontal flies was low, averaging 16.5%. Ignoring these flies, to consider only those alighted head-up or head-down on predominantly vertical surfaces, there were sometimes significant but slight changes in the percent head-up from one experiment to another, for no apparent reason. However, the consistent trend in all experiments was the marked effect of sex, species and bait type. The pooled data show that the percent head-up was 95–100% for either sex of *G. pallidipes* on all baits (N = 1526) and for female *G. m. morsitans* on the men (N = 424). For female *G. m. morsitans* on the ox it was 70–75% (N = 737), and for male *G m. morsitans* on any bait it dropped to 44–56% (N = 4988). It seems that many of the male *G. m. morsitans* on all baits were there to mate rather than feed. Hence, in the nutritional and ovarian studies, below, intended to study the nutritional status of feeding flies, the samples of *G. m. morsitans* examined were restricted to head-up flies, but for female *G. m. morsitans* and either sex of *G. pallidipes* the samples included flies of any head orientation.

#### Nutritional status

The fat extraction data for male tsetse caught from men were pooled to compare the overall effects of season and host on the nutritional status (Table 7). The TW was low in the hot-dry and warm-wet seasons, indicating that the flies were small at these times, consistent with the heat stress experienced by their mothers [23]. For all seasons taken as a whole, the AB% and Fat% of male flies on men were around two-thirds of the levels for flies on the ox, according with other work on *G. m. morsitans* and *G. pallidipes* in Zimbabwe [12] and on *G. m. submorsitans* in Nigeria [24] showing that savannah flies caught from humans are in particularly urgent need of a blood-meal. However, for *G. m. morsitans*, which involved samples large enough to elucidate seasonal effects, the Fat% of flies on men in the hot-dry season was three times greater than in the cold-dry, and was not then significantly different from flies on the ox. Against this, the AB% of *G. m. morsitans* on men was especially low in the hot-dry season, implying virtually no residual blood-meal then, so that the flies still seemed in especially urgent need of food.

**Table 7:** Nutritional status of male tsetse caught from men or oxen at various seasons.

*Species*	*Bait*	*Season*	*N*	*TW*		*AB%*		*Fat%*	
				Mean	SE	Mean	SE	Mean	SE
***G. m. morsitans***	Men	Hot-dry	125	3.92a	0.066	16.9a	1.08	28.3a	1.50
		Warm-wet	62	3.61b	0.082	24.1b	0.92	18.6b	1.45
		Cool-damp	97	4.37c	0.065	20.6c	0.68	14.4c	0.98
		Cold-dry	63	4.63d	0.095	19.7c	0.82	9.3d	0.88
	Ox	Cold-dry	97	4.45cd	0.061	32.9d	0.64	28.1a	1.14
***G. pallidipes***	Men	All seasons	18	6.38a	0.328	16.7a	1.40	14.9a	3.47
	Ox	Cold-dry	100	7.43b	0.116	24.4b	1.00	24.9b	1.82

Means of the fatless dry weight (mg) of the thorax, legs and head (TW), and the fatless abdominal weight (AB%) and fat weight (Fat%) as percentages of the TW, together with the standard error (SE) of the means, for various samples sizes (N) of *G. m. morsitans* and *G. pallidipes* caught probing or alighted head-up on men and an ox at various seasons. In any one column for any one species, means not associated with the same letter differ at P<0.05.

There was no significant effect of location on the nutritional state of male tsetse probing humans. For example, with the pooled seasonal data for *G. m. morsitans*, the mean Fat% was 19.3 (SE = 1.26) for 161 males in woodland, 19.8 (1.18) for 100 near the station buildings, and 19.2 (0.89) for 86 inside the buildings. The means of AB% for these samples were 19.1 (0.69), 19.4 (0.65) and 18.6 (0.91), respectively.

#### Age

**Figure 1:**
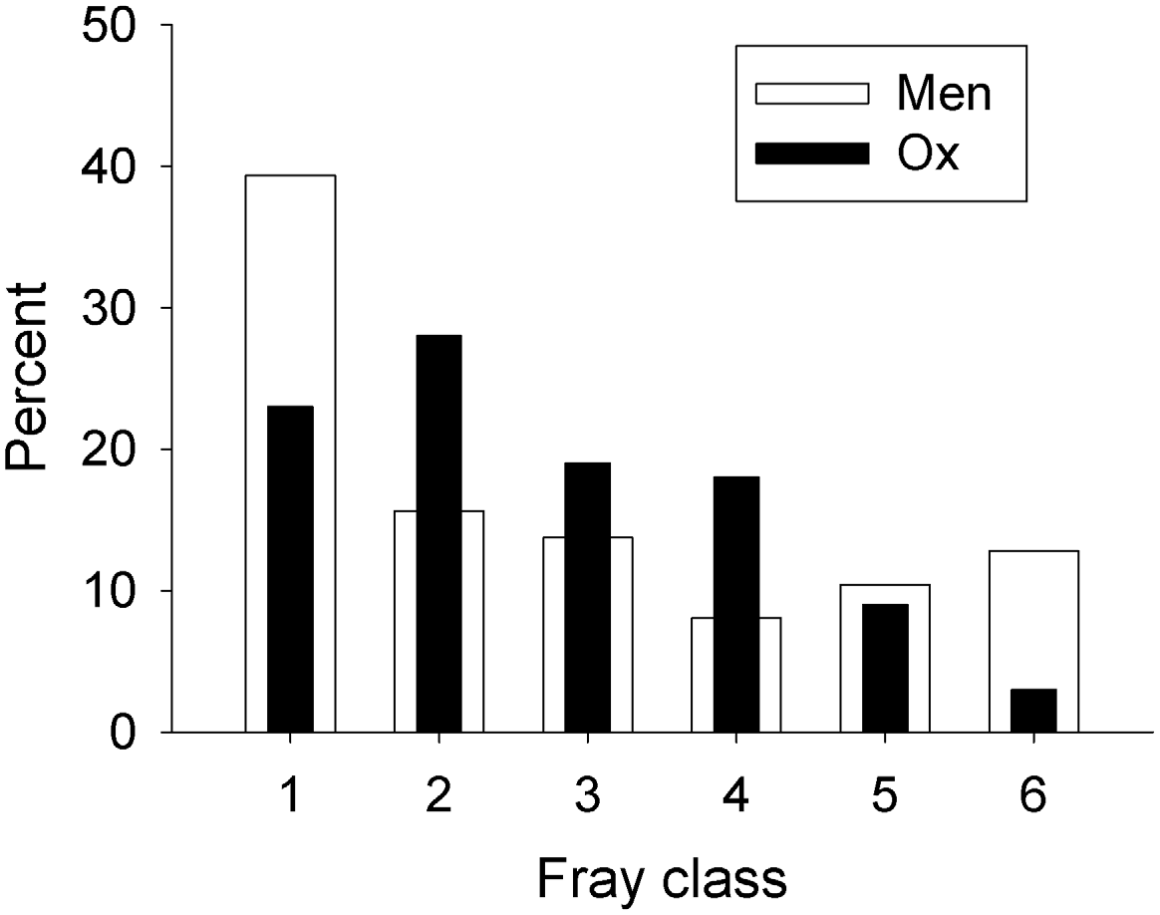
Wing fray classes of male *G. m. morsitans* caught from men and ox. Percent distribution of wing fray classes of male *G. m. morsitans* caught from men (N = 211) and ox (N = 100).

The wing fray of male *G. m. morsitans* in the above samples for nutritional studies indicated that the proportion of very young flies, *i.e.*, fray class 1, at all seasons and locations was higher than for flies on the ox. Hence the samples on men were pooled over all seasons and locations. The results (Fig. 1) show a significant (*P*<0.001) difference between the distributions, although this was due not only to the men catching many young flies, but also to their catching more of the extremely old flies, *i.e.*, fray classes 5 and 6. The few male *G. pallidipes* caught appeared to show the same trend.

The distributions of ovarian categories of the many females dissected offer more reliable and objective data for age. With trap catches the percent of young flies, *i.e.*, ovarian category< = 3, in catches showed a significant effect of season. The percentages were lowest in the cold-dry season (29%, N = 94, for *G. m. morsitans;* 22%, N = 513, for *G. pallidipes*) and highest in the cool-damp season (57%, N = 35 and 39%, N = 237), respectively. These results accord with the view that the cool-damp season is a time of population growth [25].

**Figure 2:**
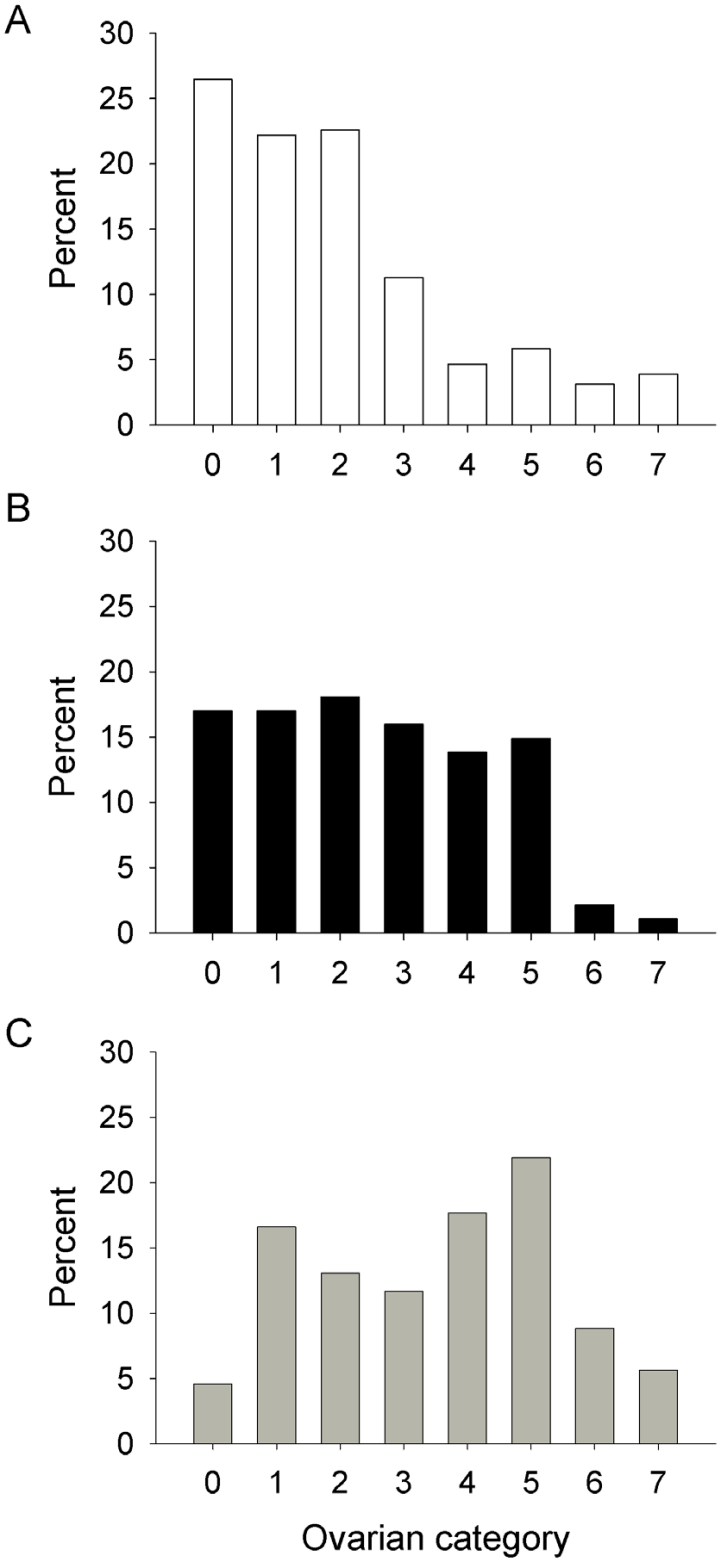
Ovarian categories of female *G. m. morsitans* from (A) men, (B) ox and (C) traps. Percent distribution of ovarian categories of female *G. m. morsitans* from (A) men, (B) ox and (C) traps. Sample sizes of 257, 94 and 283 for men, ox and traps, respectively.

Despite the seasonal variation in age structure, the over-riding indication at all seasons was that the mean age of samples from the men and from the ox with men was much lower than with trap samples, irrespective of whether the females from men were caught probing or merely alighted, and whether the men and ox were subject to any of the variations in their treatment and location. The pooled data for *G. m. morsitans* at all seasons (Fig. 2) showed that the distributions for traps differed significantly from those for the men and ox (P<0.001 in both cases), and that the distributions for the men and ox also differed significantly (P<0.01). It appeared that a relatively high proportion of the *G. m. morsitans* from men were in category 0, having never ovulated, and so were very young, although, as with males (above), there were appreciable numbers of very old flies. For *G. pallidipes* there was again a significant distinction between the generally young sample from the ox and the older sample from the traps, but since only 19 females from men were dissected their age distribution could not be compared meaningfully with those for the traps and ox, and so the detailed distributions are not depicted. However, of the 19 *G. pallidipes* from men, only three were in category 0, as against six in categories 4 to 7, *i.e.*, very old.

Unfortunately, the use of ovarian categories to assess the age of female tsetse suffers from the fact that flies in categories 4–7 include not only those that have completed 4–7 ovulations, but also those much older flies that have completed eight, or perhaps many more ovulations, i.e., flies that are exceedingly old [21]. Such very old flies can be expected to have wings in fray classes 5 and 6, characterized by much or all of the trailing edge of the wing being tattered [22]. With samples of female *G. m. morsitans* in ovarian categories> = 4, the percent of flies with wings of fray classes 5 or 6 for the sample caught probing on humans was 56% (N = 36), which was significantly and substantially greater than the 17% (N = 24) for the sample caught on the ox. It seems that exceptionally old flies formed a relatively high proportion of the old females from humans.

#### Pregnancy

**Figure 3:**
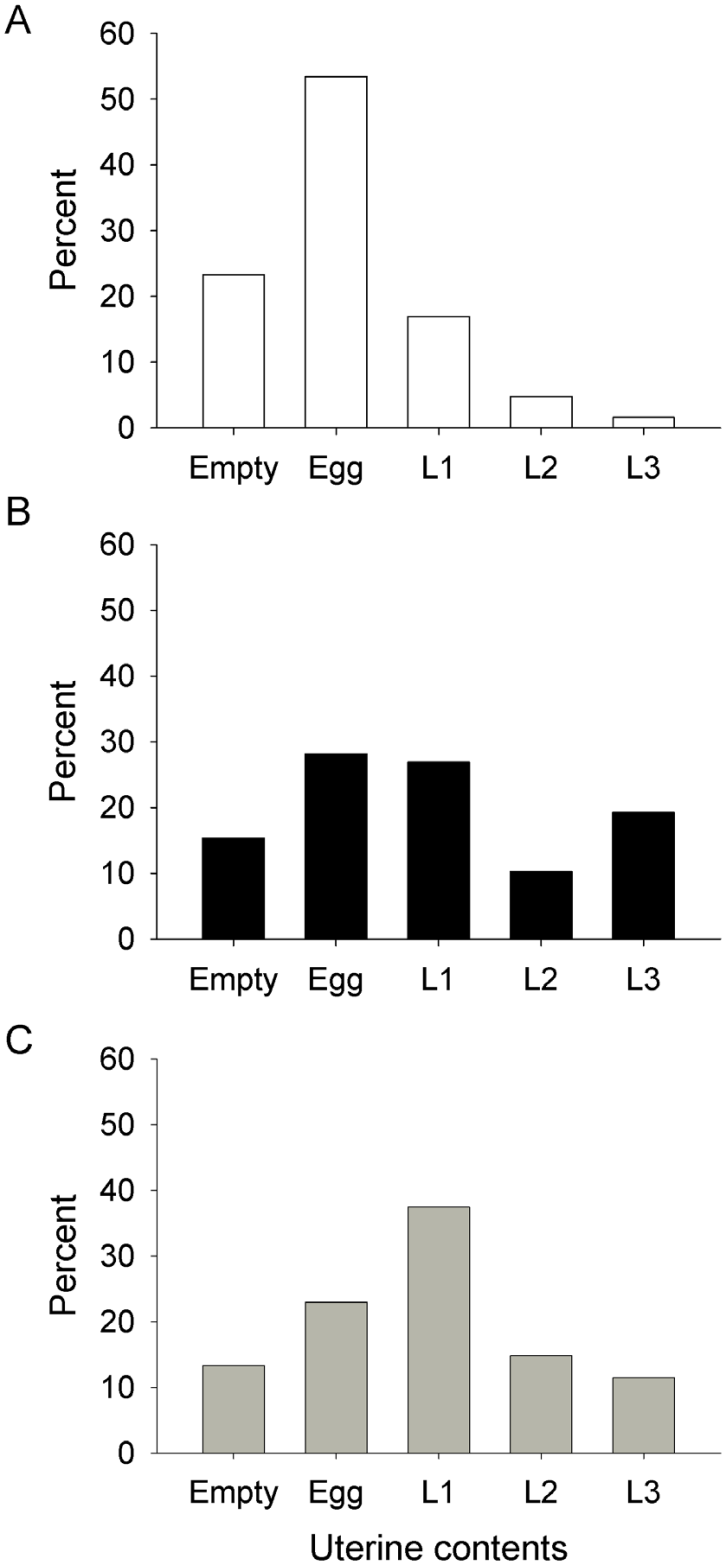
Uterine contents of female *G. m. morsitans* from (A) men, (B) ox and (C) traps. Percent distribution of uterine contents of female *G. m. morsitans* in ovarian categories>0, from (A) men, (B) ox and (C) traps. Sample sizes of 189, 78 and 270 for men, ox and traps, respectively.

**Figure 4:**
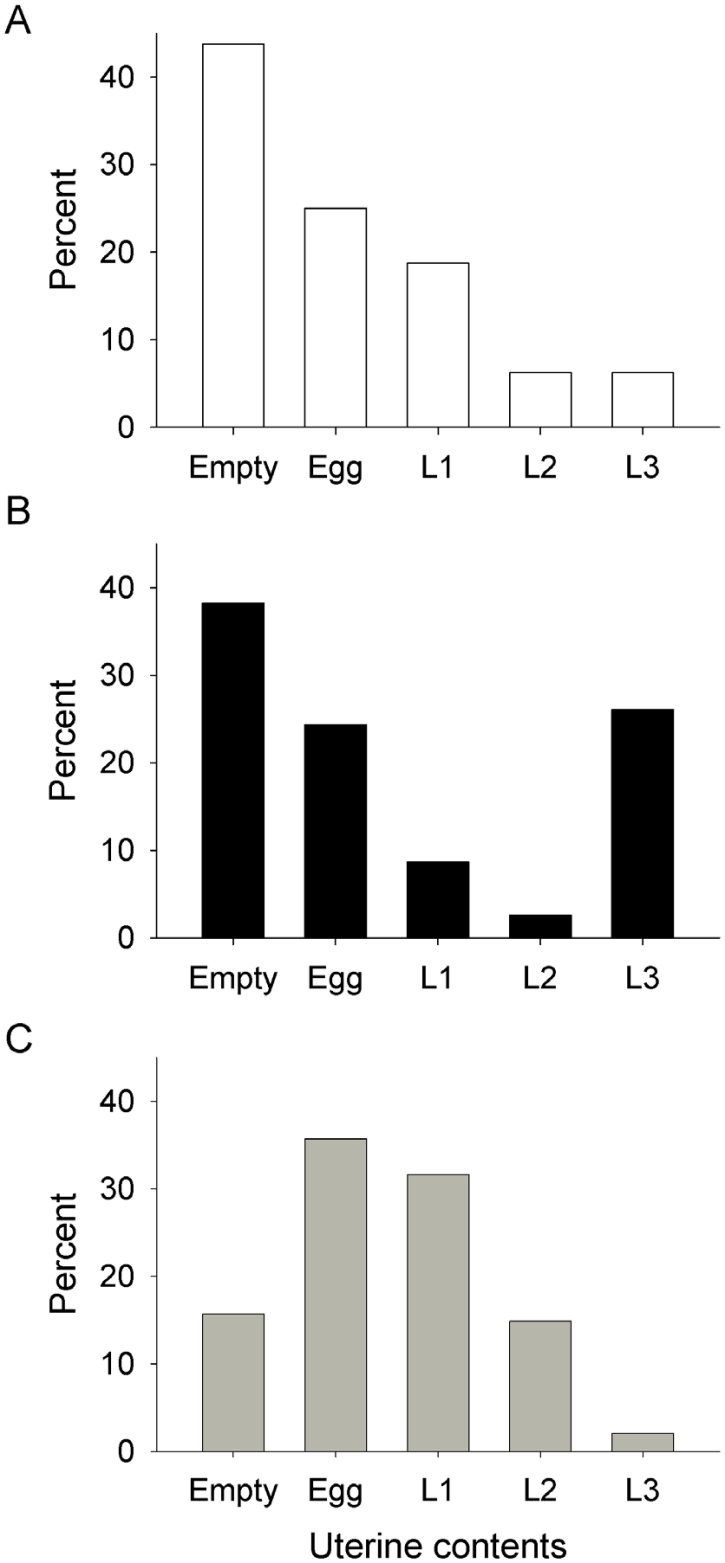
Uterine contents of female *G. pallidipes* from (A) men, (B) ox and (C) traps. Percent distribution of uterine contents of female *G. pallidipes* in ovarian categories>0, from (A) men, (B) ox and (C) traps. Sample sizes of 16, 115 and 1498 for men, ox and traps, respectively.

The uterus of a female in ovarian category 0 must be empty, by definition. For older flies the uterine contents indicate the pregnancy stage, which progresses from an egg through larval instars 1 to 3, *i.e.*, L1–L3. The distributions of the uterine contents for female *G. m. morsitans* of ovarian category>0 (Fig. 3) showed no significant difference between samples from the ox and trap. However, each of these distributions differed significantly (P<0.001) from that for the men, due primarily to the sample from men containing a very high proportion, *i.e.*, 77% (N = 189), of flies with an egg or empty uterus. The proportion remained high, at 78% (132), when the sample was restricted to females that had already completed at least one pregnancy cycle, *i.e.*, were in ovarian category 2 or above. For *G. pallidipes* (Fig. 4), by contrast, the distributions for the trap and ox differed significantly (P<0.001), due mainly to the relative absence of L1 and L2 larvae in the ox samples. The distributions for *G. pallidipes* from men were based on only 16 flies and so could not be interpreted confidently, although it was clear that these flies, like *G. m. morsitans*, were mostly in the early stages of pregnancy.

## Discussion

Our results show that for people living and working in wilderness areas there is a significant risk of being bitten within or in the vicinity of their homes and offices: ∼60% of the tsetse caught from men were while they were indoors or in the vicinity of their homes and the offices in which they worked. For humans in savannah woodland, being mobile and not in the vicinity of an ox also added to the risk of being bitten. While the tsetse caught from humans were, on average, younger than those caught from traps, about a third were old enough to have developed a mature *T. b. rhodesiense* infection. Our results imply therefore that humans confined to domestic settings and/or travelling in vehicles in tsetse-infested areas are at risk of being bitten by infective tsetse.

Many of the present results confirm long-standing knowledge of the responses of tsetse to humans. For example, it is already known that tsetse feeding on men are biased towards young males of *G. m. morsitans* with low nutritional reserves, and that mobile, rather than stationary, baits are more effective for these flies [12]. It is also known that with other savannah species, such as *G. m. submorsitans* in Nigeria, that the attack on men is much less if an ox is nearby [24]. Some of the other aspects of present results are more novel but seem to be of little practical importance. For example, the fact that *G. m. morsitans* and *G. pallidipes* probe on many parts of the body of humans contrasts with the fact that these species probe mostly on the lower body of cattle [11]. Although the sexes and species of tsetse tend to probe on different body regions of humans, the difference is not marked enough to use the location of a chancre, *i.e*., the swelling that occurs at the site of an infective bite, to determine what sex and species of fly transmitted the disease. In any event, the present data show only where flies could be caught soon after landing, not where they would eventually have fed had they not been caught and/or subject to the normal intolerance that humans show on attack.

Further aspects of present work are more intriguing. For example, the fat extraction data for male tsetse suggest that the probability of feeding on humans is not governed by low fat levels alone, but by the overall nutritional state, including the remaining blood-meal. The indication that females in the early stages of pregnancy form the majority of the older females on men accords with the fact that at this time the females need to build up their food reserves urgently, to replenish stocks exhausted at the end of the previous pregnancy [26]–[28]. It seems that for some of the older females, especially those with very heavily frayed wings, the need to feed then can become as desperate as it appears to be for those very young flies that have just emerged, with a consequent disregard of the type of host to attack. Presumably, the heavy damage to the wings limits the ability to locate hosts, so fostering extreme starvation.

The presence of old flies in the samples probing men is particularly significant, since only old flies can carry the mature infections of trypanosomes needed to transmit sleeping sickness [29]. There are three corollaries to this. First, a slight shift in the availability of the normal hosts of old tsetse could cause a marked increase in the incidence of the disease, without necessarily producing a great change in the overall number of tsetse pestering humans. Second, since many of the old flies feeding on humans can be exceptionally old they will have had time to travel relatively great distances during their lifetime, suggesting that they commonly transmit the disease in localities far removed from the place of their infection. Third, since a seemingly high proportion of the *G. pallidipes* caught from men are old, this species could be an important vector, even if its general propensity to attack men is much less than for *G. m. morsitans*.

While some of the repellence of humans is visual, there is a large olfactory component [12], [18]. Hence, the fact that the toiletries treatment did not enhance the availability of tsetse to men suggests that the repellent odour from humans does not involve residual chemicals that build up over several hours and can be removed by washing. The failure of artificial ox odour to improve the catches from men indicates that contamination of humans with residual odours from cattle would not overcome human repellence. Indeed, the clearest effect of mixing human and cattle odours at stationary baits is that the human odour reduces the response to the ox odour [12] and decreases the proportion of arriving flies that feed on the ox [30], so that the biting rate on the ox decreases greatly, especially for *G. pallidipes*. The immediate implication is that the present data for the catches from stationary men relative to a stationary ox plus men underestimate substantially the true inferiority of the men relative to an ox alone. This error of estimation could be expected to apply also when the baits were mobile.

Our results suggest that biting risk is lower when humans are stationary or wandering slowly, and when near cattle. The risk is at least ten times greater if the humans travel continuously in open vehicles at around 10 km/h. These two scenarios might typify, respectively, herdsmen tending their livestock and tourists viewing game. The risk for the tourists might be increased further by the fact that game viewing is usually conducted in the morning or late afternoon, when game animals are most readily found, and when, unfortunately but understandably, the savannah tsetse hunt the most [31], [32]. Despite these indications for substantial variations in the relative risks associated with different human activities, the absolute risk of humans getting infected in game reserves will always tend to be slight if, as usual, large numbers of the preferred hosts are available.

Present work shows that there is need for a fuller understanding of at least three topics. First, we need to know more about the risk to humans traveling in or on vehicles. For example, although it is no surprise that relatively few tsetse were caught from vehicles that moved quickly along the tarred road, at speeds far greater than the 22 km/h that tsetse could approach them [33], it is not clear why the catches from such vehicles contained high proportions of *G. pallidipes*, especially in the hot-dry season. How can we avoid or minimize the risk associated with vehicles?

Second, while trap catches of riverine species of tsetse have been used to produce credible indices of trypanosomiasis risk [34], the value of trap-based indices with the savannah species is dubious. The problem is that whereas the riverine tsetse do not seem to distinguish sharply between humans and other baits [35], [36], the savannah tsetse are much more discerning, and traps for them have been designed primarily to simulate oxen [37]–[39]. Not surprisingly, therefore, the catches of savannah species from traps and humans differ greatly in magnitude, composition and seasonal pattern, as shown by present work. Moreover, when the scarcity of natural hosts enhances the responsiveness to humans [40], or where the seemingly innate responsiveness to humans is comparatively high [24], it would be expected that the relative catches at humans and ox-like traps would change substantially. Hence, for the savannah tsetse, is it possible to design a trap that simulates a human?

Third, it was surprising that the number of tsetse caught probing men in and near the station buildings exceeded the number caught in the woodland, and that a high proportion of flies probing inside in the hot-dry season were females. This could be important because females live longer than males [23], suggesting that females could be the most likely to carry mature infections. Hence, important questions to address are: what brings various sorts of tsetse into the buildings, how is this affected by season and building design, and is the response to humans distinctive there? How can we reduce the entries and encourage exits?

Each of the above three topics is currently under investigation at Rekomitjie, but they deserve study on a wider front, especially in places where the relative abundance of humans and other hosts is different, and with a range of species showing distinctive degrees of innate responsiveness to humans.
